# New Perspectives in Modulating the Entero-Insular Axis in Pediatric Obesity

**DOI:** 10.3390/ijms26136143

**Published:** 2025-06-26

**Authors:** Loredana-Maria Dira, Loredana-Maria Marin, Simona-Georgiana Popa, Cristina-Elena Singer, Carmen-Simona Cosoveanu, Ionut Donoiu, Andreea-Loredana Golli

**Affiliations:** 1 Department of Pediatrics, University of Medicine and Pharmacy, 200349 Craiova, Romania; loredanamaria.dragan@yahoo.ro (L.-M.D.); cristina.singer@umfcv.ro (C.-E.S.); simona.cosoveanu@umfcv.ro (C.-S.C.); 2 Department of Pharmaceutical Physics, Faculty of Pharmacy, “Carol Davila” University of Medicine and Pharmacy, 020956 Bucharest, Romania; loredana-maria.marin@umfcd.ro; 3 Department of Diabetes, Nutrition and Metabolic Diseases, University of Medicine and Pharmacy, 200349 Craiova, Romania; 4 Department of Cardiology, University of Medicine and Pharmacy, 200349 Craiova, Romania; ionut.donoiu@umfcv.ro; 5 Department of Public Health and Healthcare Management, University of Medicine and Pharmacy, 200349 Craiova, Romania; andreea.golli@umfcv.ro

**Keywords:** obesity, pediatric population, entero-insular axis, Glucagon like peptide-1 receptor agonists

## Abstract

A growing global trend of adult obesity and the increasing prevalence of overweight/obesity in children indicate a higher risk in the future of adult diseases related to obesity. Current anti-obesity medications regulate appetite and metabolism by acting either in peripheral tissues or in the central nervous system. On the other hand, subsequent weight regain is a typical response to weight loss methods, and there is little evidence that current anti-obesity medications can help maintain long-term weight loss without causing a range of undesirable side effects. The combination of anti-obesity drugs targets multiple molecular pathways and structures in the central nervous system that are involved in weight regulation. This systematic review involves trials performed in pediatric populations, published up to 2025 and systematically searched on the ClinicalTrials.gov database, using “Glucagon like peptide-1 analog, Glucagon like peptide-1 receptor agonists” as the criterion for the “Intervention/treatment” category. We evaluated the entero-insular axis in pediatric patients with obesity, along with the mechanisms of action and therapeutic potential of the Glucagon like peptide-1receptor agonists. We analyzed incretin hormones and summarized the drugs approved by the Food and Drug Administration. Our objective is to identify new treatment strategies as we improve our understanding of the pathophysiology of obesity and the incretin axis.

## 1. Introduction

The World Health Organization characterizes obesity as an abnormal accumulation of fat due to a positive energy imbalance between calories consumed and calories expended [1]. Obesity and obesity-related diseases are the fifth leading cause of death globally [2]. Numerous studies have demonstrated that obesity is a complex health issue resulting from a combination of genetic, individual, and external factors (behavioral habits and socio-cultural factors) [3]. Most researchers agree that obesity is an “acquired” disease, influenced more by lifestyle factors, physical activity, and diet than by genetic components. Obesity is a condition in itself, but it can exacerbate some preexisting conditions or favor the onset of complications [4]. It is well known that excess weight is associated with an increased prevalence of cardiovascular, endocrine, metabolic, digestive, and nervous system disorders [5,6]. More and more studies have shown that phenomena related to adiposity also manifest in children [7].

A study published in 2014, conducted on a cohort of children from the municipality of Rome, highlighted that 39% of preschool children diagnosed with obesity had at least one metabolic comorbidity (insulin resistance, non-alcoholic fatty liver disease, arterial hypertension, dyslipidemia) [8].

A study published in 2017, including children between the ages of 3 and 19, examined the prevalence of cardiometabolic risk factors related to obesity, using laboratory parameters, and demonstrated that a significant percentage (92%) of those examined had at least one laboratory parameter analyzed with pathological values [9].

The development of obesity during childhood predisposes individuals to its maintenance in adulthood and is associated with long-term health complications.

In 2020, the International Childhood Cardiovascular Cohort Consortium conducted a study that concluded that approximately half of the examined adults (56%) had a history of obesity from childhood. The same study demonstrated the strong association between severe pediatric obesity and adult obesity (80% of those enrolled in the study) [10].

A meta-analysis published in 2017 by Kim J. et al. identified the association between excess weight in the 0–6 age group and the risk of metabolic syndrome in adulthood [11].

The causes of obesity’s development are complex and include socioeconomic status, parental education, school environment, physical activity, eating patterns, sleep, and screen time [12,13,14].

Several studies have indicated an important association between maternal prenatal factors such as obesity, gestational diabetes, and unhealthy behaviors during pregnancy (e.g., smoking) and excess body weight in infants (and later in children) [15,16]. Among the medical factors involved in the complex etiopathogenesis of obesity, we can mention the use of steroidal anti-inflammatories and some antibiotic therapies [7].

The body mass index (BMI) is a parameter used to identify excess weight and represents the ratio between an individual’s weight (kg) and the square of their height (m^2^). Unlike adults, where the BMI value categorizes the individual into denutrition, normal weight, overweight, or obesity, in children, the BMI category is determined by a percentile specific to their age and sex [17].

In children, overweight is characterized by BMI at or above the 85th percentile and below the 95th percentile, for children and adolescents of the same age and sex. On the other hand, obesity is defined by a BMI at or above the value of 95 for the same age and sex criteria. Severe obesity is considered when BMI ≥ 120% of the percentile for age and sex. It is estimated that obesity trends are increasing, so 57% of children aged between 2 and 19 years will be diagnosed with obesity by the age of 35 years [18]. In addition to the physical and metabolic effects, obesity in childhood and adolescence is correlated with poor psychological and emotional health, increased stress levels, depressive symptoms, and low self-esteem [19].

The healthcare costs related to obesity in the early years of life are associated with primary conditions or complications of excess weight. Observing obesity throughout life highlights how important it is to prevent and treat obesity early in life. These activities include determining the body mass index for obesity assessment, identifying individuals at high risk of obesity, promoting lifestyle optimization, and providing personalized therapy for children, adolescents, and their families.

Obesity should be treated as a chronic disease that requires intensive and long-term care, continuous monitoring, and treatment for associated comorbidities, as well as continuous access to treatment. The chronic care paradigm requires that it be provided in the context of the individual factors of the patient, including the household and family influences on the child, access to nutritious foods, and available activity spaces. The approach to obesity treatment differs based on specific individual factors. It is essential to recognize that recommendations will require modifications based on the patient’s distinct medical, familial, developmental, social, and environmental circumstances [20].

Parents play a vital role in treating childhood obesity by using strategies such as monitoring, setting limits, minimizing obstacles, managing family conflicts, and modifying the home environment [21]. Adolescence presents challenges for family care, as this phase is characterized by a typical developmental increase in the desire for independence and autonomy, despite the continued dependence on parents for various needs. Research examining certain clinical aspects related to parental involvement in the treatment of obesity in adolescents reveals inconsistent results regarding the degree of involvement and specific parental strategies that may enhance treatment efficacy [22]. It is important for therapeutic options to understand the complicated life situations of overweight children and adolescents and their families when customizing and individualizing obesity management support [23].

## 2. The Secretion of Incretins

Theories that factors generated by the intestinal mucosa in response to food consumption can stimulate the secretion of “chemicals” from the endocrine pancreas, thereby lowering blood glucose levels, were initiated in the early 1900s [24,25].

Tests have confirmed the connection between the intestines and hormone-producing cells of the pancreas, showing that oral administration of glucose increases plasma insulin levels more than intravenously administered glucose.

Incretins are peptide hormone compounds released by the gastrointestinal system after the consumption of nutrients. The two main incretins are glucagon-like peptide-1 (GLP-1) and glucose-dependent insulinotropic polypeptide, known as gastric inhibitory polypeptide (GIP) [26].

GLP-1 is a peptide hormone composed of 30 amino acids, released by intestinal L endocrine cells and primarily found in the ileum, as well as in the duodenum, colon, and rectum. This hormone is “meal-related”, with minimal plasma concentrations during fasting and significant increases during meals, especially those rich in fats and carbohydrates.

GIP is a peptide composed of 42 amino acids, released by K cells, which are specialized endocrine cells found in the duodenum and proximal jejunum. GIP is released after the consumption of nutrients, including fats and carbohydrates. The rate of nutrient absorption, rather than their mere presence in the intestine, is what triggers the release of GIP [27,28].

The primary phenomenon associated with these hormones is known as an incretinic effect. The oral delivery of glucose elicits a greater release of insulin from pancreatic β cells compared to intravenous administration, despite both techniques achieving similar glycemic levels in healthy persons [25]. This behavior is explained by the secretion of incretin hormones from the gastrointestinal tract after oral glucose administration, which does not happen with intravenous glucose.

The oral delivery of glucose stimulates GLP-1 production, but intravenous glucose treatment does not affect plasma GLP-1 levels [29], so nutrient intake is a major modulator of intestinal proglucagon gene production in humans, with fasting shown to decrease intestinal proglucagon levels, while refeeding, especially with foods rich in fiber and short-chain fatty acids, enhances gene expression in the intestinal tract [30,31,32]. The main physiological trigger for the release of physiological GLP-1 is food intake, especially those rich in lipids and carbohydrates [33]. However, it is known that hormone-producing cells that generate GLP-1 or GIP, as well as cells that generate both peptides, are present in all regions of the human small intestine [34,35]. Therefore, the production of GLP-1 from L cells is induced by a wide range of variables, including those of a neurological, endocrine, and hormonal nature. Mixed diets or single nutrients, such as glucose and other carbohydrates, fatty acids, essential amino acids, and dietary fibers, can induce the release of GLP-1 [36,37].

A study published by Herrmann C. et al. demonstrates that GLP-1 is rapidly released into the bloodstream after oral food administration, exhibiting a biphasic secretion pattern that begins with an initial phase occurring within 10 to 15 min, followed by a prolonged second phase lasting 30 to 60 min (secondary phase)37]. Considering that the predominance of L cells is in the distal intestinal segment, it is unlikely that the initial phase of GLP-1 secretion can be activated by direct contact of nutrients with the L cell. Unlike indirect processes that trigger the early release of GLP-1, the second phase of GLP-1 secretion is more likely mediated by digested foods that directly stimulate intestinal L cells [38]. Stimulating signals derived from nutrients can be sent to L cells either indirectly (through brain or endocrine mediators) or through direct contact, resulting in the early and late stages of GLP-1 production, respectively. However, since L cells appear to be present throughout the entire small intestine, it is possible that the early production of GLP-1 also occurs through direct nutritional interaction with the L cells found in the more proximal sections of the small intestine [27,33,35].

In a study published by Lauritsen K. B. et al., the relationship between the increase in plasma GIP after oral glucose administration and the significance of endogenous GIP as an incretin was illustrated [39]. They performed research including fifteen patients who had resections of different regions of the small intestine owing to Crohn’s disease or mesenteric thrombosis, in addition to ten healthy volunteers. A 50 g oral glucose tolerance test was performed alongside an intravenous glucose infusion to generate a comparable plasma glucose profile, aiming to examine the relationship between plasma GIP levels following oral glucose intake and the characteristics and length of the remaining intestinal segments, as well as the role of endogenous GIP as an incretin in humans. The magnitude of the increase in plasma GIP after an oral glucose load was strongly correlated with the length of the remaining jejunum. The incretin effect had a close correlation with the length of the residual intestine. Patients with intact ileal fragments exhibited more pronounced incretin effects compared to those with less than 150 cm of remaining jejunum and no residual ileum, although the integrated increases in plasma GIP after oral glucose administration were similar. Individuals with more than 150 cm of residual jejunum had markedly elevated plasma GIP levels and standard incretin responses when compared with healthy subjects. The production of GIP and the incretin effect in individuals with intact ileal segments were normal. Nevertheless, the incretin impact in individuals with less than 150 cm of jejunum was significantly diminished, despite a normal secretion of GIP [39].

A detailed analysis of intact incretin hormone levels after exogenous intravenous infusion in humans revealed that 40% of GIP remains intact and bioactive, compared to 20% for GLP-1. This suggests that GIP may exhibit reduced susceptibility to DPP-4 in vivo, a notion supported by the slightly longer plasma half-life of GIP compared to GLP-1 [40,41].

The increase in GIP levels in uremic patients or those with chronic kidney disease, along with the decreased GIP clearance in nephrectomized rats, suggests that the kidneys are the main system for GIP clearance. The evaluation of arteriovenous variations in GIP levels in various organs of anesthetized pigs indicated that the kidneys are the main site of GIP metabolism, while the liver and extremities also play a role in GIP extraction. The clearance rate of intact GIP and its metabolite is comparable between obese patients with type 2 diabetes and healthy individuals [42,43,44].

The enzyme dipeptidyl peptidase 4 (DPP-4) facilitates the rapid decomposition of GLP-1 and GIP into inactive metabolites. DPP-4 is a serine protease that selectively cleaves dipeptides from the amino terminus of proteins with an alanine or proline residue in the second position [35]. The half-life of intact, physiologically active GIP is approximately 7 min in healthy individuals and 5 min in patients with type 2 diabetes. GIP is also susceptible to inactivation by DPP-4. The involvement of DPP-4 in the cleavage of GIP and the formation of the inactive GIP metabolite has been unequivocally demonstrated with research on rodents, as well as on healthy and diabetic individuals, indicating that DPP-4 is the main enzyme responsible for the inactivation of GIP in vivo [27,45] Due to its rapid inactivation by DPP-4, bioactive GLP-1 has a half-life in the blood of less than two minutes. DPP-4 is significantly expressed in several organs and cell types, including the kidneys, lungs, adrenal glands, liver, intestines, spleen, testes, pancreas, and central nervous system, as well as on the surfaces of lymphocytes and macrophages. DPP-4 has been identified on the surface of endothelial cells of blood vessels and the intestinal mucosa, located directly adjacent to the secretion sites of GLP-1. Consequently, DPP-4 inactivates almost half of the GLP-1 that has already entered the portal circulation before reaching the systemic circulation [46].

The effects of GIP and GLP-1 are facilitated by the activation of G-protein-coupled receptors, located on α- and β-pancreatic cells, as well as in various organs such as the gastrointestinal tract, kidneys, central nervous system, heart, and lungs.

The mechanism of action of GLP-1 receptor agonists is largely influenced by their structural characteristics. The GLP-1 receptor has a dual-domain structure—an extracellular domain and a transmembrane domain—and interacts with GLP-1 receptor agonists, which are typically long α-helical peptides. While the transmembrane domain accommodates the N-terminal section within a deep orthosteric pocket and forms essential polar interactions with transmembrane helices (TM1, TM2, and TM3), the extracellular domain binds the peptide’s C-terminal part [47,48]. In addition to stabilizing the agonist within the receptor, this deep anchoring of important residues like His7 and Glu9 enables a structural reorganization, including an outward shift of the intracellular half of TM6, which is necessary for G-protein coupling and downstream signaling [49]. According to the molecular structure, the peptide agonist adopts an α-helical shape and is firmly positioned inside the receptor-binding pocket. These findings highlight the importance of the structural conformation of both the ligand and receptor in driving selective receptor engagement, activation, and therapeutic efficacy.

Activation of GLP-1 receptors stimulates adenyl cyclase, resulting in increased levels of cyclic adenosine monophosphate (cAMP) and subsequent activation of protein kinase A. This leads to glucose-dependent insulin secretion [26]. The molecular pathways through which GIP enhances glucose-dependent insulin secretion significantly coincide with those of GLP-1, with both GIP and GLP-1 stimulating insulin secretion in a glucose-dependent manner [50,51]. GIP reduces pancreatic beta-cell apoptosis induced by glucolipotoxicity, and it also restores insulin secretion suppressed by glucolipotoxicity.

GLP-1 inhibits the release of glucagon from pancreatic alpha cells only under hyperglycemic and euglycemic conditions, thereby reducing hepatic glucose synthesis. Glucagon production is unaffected in case of hypoglycemia, even when therapeutic amounts of GLP-1 are present. Interestingly, GLP-1’s glucagonostatic actions remain the same in diabetics, while its insulinotropic effects are significantly reduced [41]. Conversely, GIP enhances glucagon secretion, intensifies the postprandial glucagon response, and stimulates pancreatic alpha cells to produce glucagon during hypoglycemia or euglycemia, but not during hyperglycemia.

Furthermore, GLP-1 reduces pentagastrin- secretionand meal-induced gastric acid secretion. It also reduces gastrointestinal motility, aiding in the stabilization of blood glucose levels in type 2 diabetes after therapy with exogenous GLP-1, and enhances satiety, with a consequent reduction in food intake [52]. Additionally, GLP-1 has a direct influence on hypothalamic neurons, modulating energy balance and food intake [45,50].

GIP enhances blood flow in adipose tissue and also promotes the accumulation of triglycerides in adipose tissue. The anabolic effects of GIP include the promotion of fatty acid synthesis, re-esterification, insulin-mediated incorporation of fatty acids into triglycerides, stimulation of lipoprotein lipase, and attenuation of glucagon-induced lipolysis [25]. It is estimated that the incretin phenomenon contributes approximately 50–70% of the total insulin secreted after oral glucose administration [53,54].

The effects of GIP on pancreatic β cells are comparable to those of GLP. However, GIP has distinct physiological effects on extrapancreatic organs. The activity of GIP in the central nervous system can influence the growth of neural progenitor cells and behavioral changes [55].

GIP is involved in the regulation of lipid metabolism and the onset of obesity. The consumption of fats significantly stimulates the release of GIP in humans, and plasma levels of the GIP precursors are elevated in some obese individuals. The anabolic effects of GIP on adipose tissue include the stimulation of fatty acid synthesis, the insulin-mediated incorporation of fatty acids into triglycerides, and the attenuation of glucagon-induced lipolysis [56,57].

Therefore, while individuals with type 2 diabetes exhibit considerable resistance to the insulinotropic effects of exogenous GIP administration and there is no direct correlation between obesity and GIP in humans, the comparative advantages of inhibiting versus activating GIP receptor signaling must be evaluated in future therapeutic applications of GIP or its analogs.

People who are overweight/obese or are known to have type 2 diabetes have lower postprandial GLP-1 levels detected [58,59,60,61]. Compared to individuals with normal weight, those with overweight and patients with type 2 diabetes have comparable rates of GLP-1 elimination, and the observed decrease in GLP-1 levels is most likely the result of reduced GLP-1 production. The stimulation of glucose-dependent insulin secretion is just one of the many biological actions of GLP-1 receptor agonists in the pancreas, improving glucose sensitivity in glucose-resistant β cells, thereby increasing their ability to detect and respond to glucose levels [62,63,64].

Additionally, GLP-1 promotes the release of somatostatin and reduces glucagon. The stimulatory influence of GLP-1 on somatostatin secretion is likely due to direct contact with GLP-1 receptors on pancreatic alpha cells that secrete somatostatin. In individuals with type 1 diabetes who are fasting and do not have the capacity to secrete insulin from their beta cells, the inhibitory impact of GLP-1 on glucagon production has also been observed, suggesting that the glucagonostatic action of GLP-1 may be mediated without the help of endogenous insulin levels [65,66,67].

There are two ways in which GLP-1 agonists can conserve beta-cell mass: firstly, by directly interacting with GLP-1 receptors, activating intracellular signaling pathways that promote beta-cell proliferation and neogenesis and decrease apoptosis; and secondly, by indirectly protecting cells from a harmful metabolic environment by reducing elevated blood glucose levels and free fatty acids.

Exogenous administration of GLP-1 also contributes to the reduction in postprandial glycemic peaks, both in patients with type 2 diabetes and in those with type 1 diabetes, through the effect of delaying the transit of nutrients from the stomach to the small intestine [68]. Based on experimental data, the inhibitory effect of GLP-1 on gastric emptying and acid secretion seems to be vagally mediated, with GLP-1 receptors located in the central nervous system and/or on vagal afferent fibers, transmitting sensory signals to the brainstem. These glycemic effects represent a potential explanation for the correlation between exogenous administration of GLP-1 receptor agonists and improved endothelial function in patients with type 2 diabetes [26].

Plasma concentrations of GIP are normal or elevated in individuals with type 2 diabetes, while meal-induced serum levels of GLP-1 are slightly but significantly reduced in patients with impaired glucose tolerance and those with type 2 diabetes [58].

On the other hand, although the glucoregulatory effects of GLP-1 remain intact in individuals with type 2 diabetes, the immediate insulinotropic impact of native GIP is significantly reduced in diabetic patients [69].

Nauck M. et al. refer to the “incretin effect” in healthy adults as the secretion of insulin in response to orally administered glucose, which is nearly 50% greater due to incretins produced by the intestine. This difference in the insulin response between oral and intravenous glucose loading is largely attributed to these incretins [70]. Knop F. K., similar to Nauck M. et al., demonstrated that in adults with type 2 diabetes, the incretin response is reduced [71,72].

Calanna S. et al. investigated the entero-insular axis in a cross-sectional study that included 129 obese and 29 normal-weight subjects [73]. Based on a homeostasis model assessment of insulin resistance (HOMA-IR), obese subjects were classified as metabolically healthy obese (lower tertile of HOMA-IR) or at-risk obese (upper tertile of HOMA-IR). Participants with metabolically healthy obesity, compared with at-risk obese subjects, had lower blood levels of glucose, insulin, and C-peptide at baseline and at 30, 60, 90, and 120 min during the 75 g oral glucose tolerance test (OGTT) and lower baseline plasma glucagon levels and appropriate glucagon suppression after oral glucose loading, changes that might be explained by the impaired insulinotropic activity of GIP, despite higher plasma GIP levels and impaired postprandial secretion of GLP-1 in subjects with at-risk obesity. Metabolically healthy obese participants, like those with normal weight, had a lesser diabetogenic profile owing to a greater disposition index and an unaltered entero-insular axis. Obese persons at risk have raised GIP levels, which may contribute to increased glucagon secretion and insufficient glucagon responses following glucose loading, resulting in disturbed glucose homeostasis. The study’s significant finding was that obese persons with a normal metabolic profile and those with normal weight have a comparable disposition index, indicating that beta-cell function is preserved. In contrast, at-risk obese people’s beta-cell function is insufficient to compensate for insulin resistance. In metabolically healthy obese participants, however, both baseline glucagon levels and glucagon suppression during the OGTT were comparable to those of control subjects but distinct from those of at-risk obese persons. Since GIP has been shown to have glucagonotropic effects in humans, greater levels of GIP in at-risk obese persons may contribute to increased basal glucagon levels and poor glucagon suppression following glucose loading [73].

## 3. Particularities of the Incretin Axis in Children

Disruptions in the production of pancreatic and intestinal hormones are noted in cases of obesity and may correlate with the degree of insulin resistance, prompting inquiries into the involvement of these hormones in the onset of obesity throughout childhood and adolescence.

Children and adolescents with obesity and insulin resistance have increased levels of fasting plasma glucagon and GLP-1, increased glucagon, and diminished GLP-1 responses during the OGTT, corresponding with reduced insulin sensitivity and β-cell function. Children and adolescents with obesity and adequate insulin sensitivity have distinct variations in glucagon and incretin production, indicating opportunities for tailored therapies.

Similar to the study conducted by Calanna S. in an adult population, Stinson S. E. et al. evaluated the incretin effect in a cohort of 80 children and adolescents from the Danish Childhood Obesity Data and Biobank—HOLBAEK study (ClinicalTrials.gov identifier number NCT00928473), aged 7 to 17 years, grouped based on BMI and Matsuda index into three categories: obese with insulin resistance, obese with normal insulin sensitivity, and normal-weight controls [73,74]. An OGTT (1.75 g/kg, maximum 75 g glucose) was performed in all subjects included in the study, and the plasma levels of glucose, insulin, glucagon, GIP, and GLP-1 were assessed during fasting and at 30 and 120 min after glucose loading. Subjects with obesity and insulin resistance had significantly higher fasting plasma glucose, insulinemia, glucagon, and GLP-1 levels and similar fasting GIP values compared with those with obesity and normal insulin sensitivity. Obesity with normal insulin sensitivity was characterized by lower glucagon levels and similar levels of glucose, insulinemia, GLP-1, and GIP compared with normal weight, while obesity with insulin resistance was characterized by higher levels of glucose and insulinemia and similar levels of glucagon, GIP, and GLP-1 compared with normal weight.

Furthermore, children and adolescents with obesity and insulin resistance had elevated glucagon levels and diminished GLP-1 responses during the OGTT, in comparison to their counterparts with obesity and insulin sensitivity or those with normal weight. In contrast to obese and insulin-resistant children and adolescents, persons with obesity and insulin sensitivity had no substantial variations in hormonal responses when compared to normal-weight controls. Elevated glucagon and diminished GLP-1 responses were correlated with impaired insulin sensitivity and β-cell function. This study’s results indicate that insulin resistance is associated with obesity-related alterations in glucagon and GLP-1 production, potentially impacting future illness risk [75].

In a longitudinal study conducted by Galderisi A., the effect of incretins on cell function was studied in a group of adolescents with obesity and prediabetes, demonstrating that the significantly increased incretin effect serves as a protective factor for beta-cell function over time, compared to young individuals who exhibit a low incretin effect characterized by a low-insulin-sensitivity phenotype [76].

Since the studied group included young people aged 15–19, the research proposed the significant effect of incretins on beta-cell function in the long term, highlighting the need for therapeutic interventions designed to maintain the effect of incretin during childhood.

Energy metabolism and the pathogenesis of metabolic disorders are influenced by the entero-insular axis in pediatric patients. For the therapy to be effective, it is necessary to know how it behaves depending on age and metabolic status. In children and adolescents, the entero-insular axis’s function is maturing, which can influence the incretin response and insulin secretion. Rapid growth and increased metabolic needs during this period can affect insulin sensitivity. In obese children, the release of GIP and GLP-1 may be altered, contributing to insulin resistance and hyperinsulinemia. In children with type 1 diabetes, the production of incretins may be normal, but the pancreatic response is compromised due to the destruction of β cells. In pediatric type 2 diabetes, insulin resistance and decreased incretin response can worsen glycemic control [73].

Studies on the entero-insular axis in children are still limited, but the available research underscores the importance of this axis in glucose homeostasis and energy metabolism.

In obese children, the function of the entero-insular axis can be altered, contributing to insulin resistance. There are also data regarding its role in metabolic disorders associated with anorexia nervosa and pediatric type 2 diabetes, indicating the potential for personalized therapeutic interventions. Michaliszyn S. F. and Aulinger A. B. have highlighted that the effect of incretin diminishes as hyperglycemia progresses towards overt diabetes, both in childhood and in adults [75,77].

Identifying the metabolic phenotype of young people with obesity and prediabetes, as well as their disease history in relation to beta-cell function and the incretin effect, is critically important in developing diabetes prevention strategies that target the metabolic determinants of the young.

The entero-insular axis is crucial in generating satiety signals. Therefore, disturbances of this axis can affect the progression of anorexia nervosa [78]. The purpose of the study that was conducted by Tomasik P. J. et al. was to investigate the function of the hormonal component of the entero-insular axis in female adolescents aged 13 to 16 years who suffer from anorexia nervosa [78]. For the purpose of their experiment, they looked at thirteen females who had been diagnosed with anorexia nervosa and ten girls who were healthy. Additionally, a standard meal test and an oral glucose tolerance test were performed on each of the girls, blood samples being collected fasting and at 15, 30, 60, and 120 min after stimulation. The concentrations of these peptides in fasting and postprandial conditions, along with the cumulative values, were evaluated. Fasting insulin levels were significantly elevated in the group of women with anorexia nervosa compared to the control group. Insulin production in girls with anorexia was considerably lower during the oral glucose tolerance test compared to postprandial levels. The production of glucagon in both tests was increased in the group of women with anorexia compared to the control group. The average levels of pancreatic polypeptide and cholecystokinin in the anorexia group were significantly increased compared to the control group, but only after the test meal. The values of GIP in both assessments were significantly higher in anorexic girls compared to the control group. In contrast, the average values of GLP-1 production in both assessments were significantly higher in the control group than in the anorexic girls [78].

The significance of the entero-insular axis in the control of energy balance seems to be underestimated. GIP and GLP-1, as incretin drugs, directly affect insulin production and indirectly regulate blood glucose levels. Both glucose and insulin are recognized as variables of satiety [79,80]. GIP, cholecystokinin, and GLP-1 also affect the release of other pancreatic hormones—glucagon and pancreatic somatostatin—significantly contributing to the control of the body’s metabolic balance. Additionally, GIP and GLP-1 function as enterogastrones that prevent gastric emptying, thereby affecting the feeling of satiety produced by the mechanoreceptors in the gastric walls [81,82]. The observed findings highlight that postprandial entero-insular axis hormone production in children with anorexia nervosa may exacerbate the progression of this disorder. The effects of these hormones are most evident after the oral intake of glucose.

## 4. Glucagon Like Peptide-1 Receptor Agonists in the Treatment of Obesity in Children

### 4.1. Data Analysis and Extraction Strategy

We systematically searched the ClinicalTrials.gov database for studies and trials published up to and including the date of 31 March 2025, using the keyword “Obesity” as the criterion for the “Condition/disease” category and the keywords “GLP-1 analog, GLP-1 receptor agonists” as the criterion for the “Intervention/treatment” category. To select relevant trials performed on pediatric populations, we used supplementary filters such as Child (birth—17) as eligibility criteria. Hypothalamic obesity, obesity associated with Prader–Willi syndrome, and the impact of GLP-1 agonist therapy on individuals with obesity and type 2 diabetes were used as exclusion criteria.

To identify relevant studies, we screened the PubMed database, using the keywords “Obesity, GLP-1 receptor agonists/analogs, incretins” in children and adolescents or pediatric populations. A reverse search was performed, which consisted of analyzing the references from all selected original research studies, meta-analyses, and systematic reviews, for the identification of other additional studies.

In total, we identified 33 studies related to GLP-1 receptor agonist therapy in children and adolescents with obesity, of which 8 studies passed the screening because they involved pediatric populations and the use of GLP-1 receptor agonists.

### 4.2. Liraglutide

Liraglutide is a once-daily subcutaneous agonist of the glucagon-like peptide-1 (GLP-1) receptor. By suppressing glucagon release, increasing glucose-dependent insulin secretion, decreasing the stomach’s emptying rate, and inhibiting orexigenic neurons, it has anorexigenic and metabolic actions [51]. In the management of metabolic disorders, liraglutide displays a significant improvement. It is frequently used in contemporary medical care due to its efficiency in controlling glucose levels and weight reduction, as well as its generally favorable safety profile. However, administration requires close supervision, while therapy needs to be individualized to each patient.

In addition to BMI reductions, liraglutide has demonstrated substantial weight reduction and enhanced metabolic indicators, including blood glucose levels, triglycerides, and cholesterol [83,84]. Liraglutide-induced weight loss can persist for at least a year, based on preliminary data, especially if treatment is accompanied by ongoing changes in lifestyle. Patients must continue focusing on healthy habits after therapy ends, since post-medication rebound weight gain may occur [85].

Although usually well tolerated, there have been some reported rare but serious side effects, including renal impairment, gallbladder disease, and pancreatitis, which emphasize the significance of long-term safety monitoring [86].

For the management of obesity, liraglutide is frequently recommended when changes in diet and physical activity are not sufficient. It needs to be an essential component of a comprehensive treatment plan that involves behavioral therapy and family support. Evaluating the potential risks and adverse effects is essential, especially for children who already have diseases such pancreatitis or digestive disorders.

### 4.3. Semaglutide

Semaglutide is a long-acting agonist of the GLP-1 receptor. By promoting satiety, reducing appetite, and reducing calorie intake and potential food consumption, it reproduces the effects of GLP-1. It additionally lowers blood glucose by reducing glucagon release and increases insulin secretion, in a dependent-glucose manner [83,84,87,88].

Abdominal pain, fatigue, and headaches are some of the adverse effects reported. While serious side effects are rare, cases including pancreatitis and gallbladder disease have been noted, highlighting the necessity of careful monitoring [89].

According to preliminary data, long-term use may help maintain weight loss and promote additional improvements in metabolic health. The management of childhood obesity has advanced significantly with semaglutide; nevertheless, further research is required to confirm its long-term efficacy and safety in children, as well as to determine the most appropriate approach for every patient.

### 4.4. Exenatide

Inhibiting glucagon secretion, delaying stomach emptying, reducing food consumption, and improving glucose-dependent insulin secretion from pancreatic beta cells are several of the advantages of exenatide, a GLP-1 receptor agonist [90]. It is uncommon to administer exenatide monotherapy for overweight children. Exenatide has been associated with modest weight loss and improved glycemic control in pediatric populations [91,92]. Gastrointestinal side effects, including nausea, vomiting, diarrhea, and abdominal pain, are commonly reported in children and adolescents [91,92]. Concerns remain regarding the safety profile of GLP-1 receptor agonists in this population, particularly the potential risk of pancreatitis.

Unlike other GLP-1 receptor agonists such as semaglutide and liraglutide, exenatide is not approved by the Food and Drug Administration for weight management in children and adolescents [93,94]. Most pediatric research on exenatide has focused on patients with type 2 diabetes rather than isolated obesity. Consequently, the use of exenatide for childhood obesity remains limited, with weight loss effects generally being less pronounced compared to other agents in its class.

Table 1, Table 2 and Table 3 provide an overview of the study characteristics, clinical efficacy, and side effects of GLP-1 receptor agonists, respectively, in pediatric patients with obesity.

BMI, body mass index.

## 5. Discussion

GLP-1 receptor agonists are becoming a promising pharmaceutical alternative for treating childhood obesity, especially in adolescents 12 years of age and older. Such drugs, which initially were developed to help control blood glucose levels in people with type 2 diabetes, have a variety of metabolic effects, such as promoting the secretion of insulin, suppressing the release of glucagon, delaying the process of stomach emptying, and increasing feelings of satiety [83,84]. Therefore, GLP-1 receptor agonists provide a useful additional approach for weight loss in young individuals, considering the insufficient long-term effectiveness of lifestyle treatments alone [83].

Mild-to-moderate adverse effects are relatively common and vary depending on the specific GLP-1 receptor agonist used, although the overall risk of serious adverse events remains uncertain. Evidence on the impact of GLP-1 receptor agonists on psychological outcomes, including anxiety and depression, is currently limited. Furthermore, the safety and efficacy of these agents in children under 12 years of age remain unclear, with most clinical data being derived from studies involving adolescents aged 12 and older. In light of these limitations, the current guidelines conditionally recommend the use of GLP-1 receptor agonists in pediatric populations, based on evidence rated as very low to low in certainty [99].

The safety and efficacy of GLP-1 RAs in overweight or obese children and adolescents under the age of 18, without diabetes mellitus, were assessed by Katole et al. [83] in a meta-analysis. Weight-related outcomes in populations with BMI > 30 kg/m^2^ were the primary objective of the analysis, which included randomized controlled studies comparing GLP-1 receptor agonists to standard treatment or placebo. By increasing insulin secretion, inhibiting glucagon, and decreasing the consumption of calories through decreased gastrointestinal motility and central anorectic processes, GLP-1 receptor agonists like semaglutide have anti-obesity benefits. Semaglutide outperforms liraglutide and exenatide in terms of weight reduction effectiveness among these medications. Given the challenges associated with sustaining lifestyle interventions over the long term and the limited availability of pharmacotherapeutic options, the recent Food and Drug Administration approval of semaglutide for adolescents aged 12 years and older underscores its significant clinical value. Analysis results demonstrate that GLP-1 receptor agonists, particularly semaglutide, successfully lower weight and BMI in obese adolescents who are not diabetic. The gastrointestinal side effects of these agents are similar, indicating that they may enhance health outcomes and decrease the complications associated with obesity in this population.

Ryan P. M. et al. investigated the efficacy, safety, and related limitations of utilizing GLP-1RAs in obese children and adolescents [84]. GLP-1 receptor agonists were associated with moderate but significant reductions in body weight, BMI, and BMI z-score across nine studies with a total of 574 subjects. Particularly in groups formed exclusively of children with insulin resistance, the treatment proved to be beneficial, and its efficacy was increased when combined with lifestyle modifications. There were no significant treatment-emergent adverse events, although nausea was the most commonly reported adverse event. According to the results, GLP-1 can improve the cardiometabolic profile of obese children and adolescents in a moderately safe manner.

Therefore, comparing **semaglutide** to other agents in its class, it has shown the most significant effect on body weight reduction in adolescents, with a significant effect on BMI z-score and a moderate increase in health-related quality of life [99].

The therapeutic efficacy of semaglutide in treating adolescents with obesity is supported by clinical studies. In comparison to lifestyle modifications alone, once-weekly administration of 2.4 mg of semaglutide in combination with lifestyle modifications resulted in significant reductions in BMI, BMI z-score, and total body weight, according to the STEP TEENS randomized controlled trial, which included 201 participants between the ages of 12 and 17 years [89]. Clinically significant weight loss was attained by a significant percentage of teenagers, with reductions ranging from 5% to 20%. The trial demonstrated that triglyceride levels significantly decreased and low-density lipoprotein cholesterol moderately decreased, along with improvements in anthropometric parameters. Systolic blood pressure, total cholesterol, and high-density lipoprotein cholesterol all showed modest but positive improvements. Despite these benefits, semaglutide treatment was linked to a significant rate of side effects, which affected 82% of individuals in the placebo group and 79% of patients in the semaglutide group. The most frequently reported side effects were gastrointestinal disorders, including nausea, vomiting, and diarrhea. All five cases of cholelithiasis only occurred in the semaglutide arm, and 11% of people receiving semaglutide experienced serious adverse effects compared to 9% in the group receiving the placebo. Furthermore, the semaglutide group showed a slightly greater rate of treatment ending due to adverse events (5%) compared to the placebo group (4%) [89]. The STEP TEENS trial has a number of limitations that may have an impact on how its findings are interpreted. One notable drawback is the relatively short duration of both the treatment and follow-up periods. The treatment period was 68 weeks, with a 7-week follow-up. Given that semaglutide’s effects have been demonstrated to last for more than two years in adults, a longer treatment duration would have aided in determining how long the benefits would persist. Likewise, given the little increase in BMI observed between weeks 68 and 75, a longer follow-up period would have enabled the better evaluation of the outcomes following medication discontinuation. The trial population’s composition is another significant drawback. Some racial and cultural groups were underrepresented in the study, and more women than men were included.

Another studied agent, liraglutide, has also been associated with significant BMI reductions in adolescents with obesity [49,86,95,97,100]. It increases insulin secretion, suppresses glucagon release in a glucose-dependent path, and helps in weight loss by reducing energy intake and hunger [84,97].

Liraglutide significantly reduced BMI in teenagers aged 12 to less than 18 years, according to data obtained by Kelly A.S. et al. [86]. In particular, liraglutide reduced the baseline BMI by ≥5% in 43.3% of participants, compared to just 18.7% in the placebo group, and by ≥10% in 26.1% of patients, compared to 8.1% in the placebo group. These results highlight the possibility of liraglutide as a successful pharmaceutical treatment for childhood obesity. An important limitation of interpreting these results is that the percentage of samples collected for pharmacokinetic analysis that had liraglutide concentrations below the lower limit of quantification increased near the end of the treatment period. This pattern suggests a decline in actual drug adherence, despite self-reported or recorded adherence rates remaining high (>80%).

The efficacy of liraglutide, when combined with lifestyle modifications at doses up to 3.0 mg/day, was examined in a post hoc analysis of the SCALE Teens trial. Teenagers between the ages of 12 and under 18 who had a BMI of at least 30 kg/m^2^ were included in the study. Liraglutide was linked to considerably higher BMI reductions (≥5% and ≥10%) than the placebo, regardless of baseline variables such sex, age, ethnicity, pubertal status, glycemic control, symptoms of depression, or level of obesity. Participants with increased glucose levels experienced a quantitatively decreased treatment benefit, even though baseline hyperglycemia had no discernible impact on outcomes. In adolescents with obesity, liraglutide showed overall clinically relevant efficacy, with early response acting as a crucial predictor of long-term benefit. However, the insufficient number of participants in certain groups and the trial’s initial design, which limited the power to detect subgroup differences, constrained the study [49]. When interpreting the results, it is important to take into account the limitations of the SCALE Teens trial’s secondary analysis. The study was not statistically powered to detect variations in treatment effects within the evaluated subgroups, since the analysis was not originally planned as part of the trial design. Although the pharmacokinetic results suggested a possible decrease in adherence toward the conclusion of the treatment term, the study found an overall good adherence rate of 81% among subjects treated with liraglutide.

Mastrandrea D. et al. [97] extended the focus to younger children by assessing the safety, tolerability, pharmacokinetics, and pharmacodynamics of liraglutide in obese children between 7 and 11 years of age. Subcutaneous injections were administered once daily to the participants, with doses ranging from 0.3 mg to a maximum of 3.0 mg or the highest tolerated dose. There were no additional safety concerns identified, and the safety and tolerability profile of this pediatric group was consistent with that of adolescents and adults. Although the liraglutide group’s BMI z-score was much lower than that of the placebo group, the body weight decrease was not statistically significant, most likely as a result of the trial’s short length and small sample size. Liraglutide showed a good safety profile and the potential to be effective in lowering obesity in this younger pediatric population, in spite of these drawbacks. Group variability, an unequal sex distribution in the placebo arm, and a short trial period to evaluate long-term safety or cardiovascular consequences are some of the study’s weaknesses. Several limitations and possible biases are acknowledged in the study on liraglutide in obese youngsters. The treatment groups’ heterogeneity is one downside; more male participants in the placebo group meant that they were, on average, taller and heavier than those in the liraglutide group. When comparing the results of the two groups, this imbalance may cause bias. Additionally, fewer female children were included in the study, due to the exclusion criteria related to puberty, which may restrict the findings’ applicability to the larger community of obese children. The trial’s short duration—roughly seven weeks—is another important drawback. The trial’s brief duration prevented a thorough understanding of the exposure–response link, even though exploratory pharmacokinetic analysis indicated a relationship between drug exposure and response.

Exenatide, another GLP-1 receptor agonist, has been shown to be effective in stimulating weight loss and is approved for the treatment of type 2 diabetes mellitus in adults. By promoting postprandial insulin secretion in a glucose-dependent path, it improves glycemic control. Exenatide can be administered once a week due to its extended-release profile. The potential of exenatide in treating childhood obesity has also been investigated.

Weghuber et al.’s randomized experiment evaluated the impact of exenatide on obese adolescents [98]. The study focused on teenagers between the ages of 10 and 18 who had been diagnosed with obesity. The purpose of this study was to evaluate this long-acting GLP-1 analog’s efficacy, safety, and tolerability in obese pediatric patients. According to the results, extended-release exenatide is generally well tolerated and has been associated with slight BMI decreases in obese adolescents, as well as improvements in lipid profiles and glucose tolerance. Furthermore, the group treated with exenatide experienced gastrointestinal adverse events more frequently. The study’s small sample size could restrict how broadly the findings can be applied, and more investigation is required to assess treatment adherence, safety, and long-term efficacy.

Identifying the determinants of weight loss responsiveness to exenatide in adolescents with severe obesity was also a key objective of the study conducted by Nathan et al. [101]. Two clinical studies that evaluated the impact of exenatide on weight loss were combined. Teenagers between the ages of 12 and 19 who had been diagnosed with severe obesity were included in the target population. For the first month, 5 mcg twice daily of exenatide was administered subcutaneously. For the next two months, the dose was increased to 10 mcg twice daily. In line with results from earlier studies, exenatide treatment resulted in a BMI decrease of roughly 3–5% when compared to placebo or control groups. Compared to a placebo, the combined analysis showed a statistically significant drop of −3.42% in absolute BMI at three months. Notably, when treated with exenatide instead of a placebo, subjects who reported higher baseline hunger scores saw noticeably larger reductions in BMI (−4.28% vs. 1.02%, *p* = 0.028). In subgroup analysis, the BMI of female participants was significantly lower than that of the placebo group (*p* < 0.001), whereas the male subgroup showed no statistically significant difference. The results show that the change in BMI following three months was significantly predicted by sex and baseline hunger levels. In particular, the best baseline predictors of a positive weight loss response to exenatide were female sex and increased self-reported hunger. These findings indicate that unique patient factors could influence therapy responsiveness and direct customized therapeutic approaches for adolescent obesity.

Regarding the therapeutic modulation of GIP levels and activity, unlike GLP-1, which is targeted by multiple drugs, and despite numerous studies conducted in humans that utilized GIP receptor agonists (GIP(1–42) and GIP(1–30)NH2) and the GIP receptors an-680 tagonist (GIP(3–30)NH2) to elucidate the acute physiological and pathophysiological functions of the GIP system, there is currently no therapeutic option available that acts exclusively through GIP [93]. The development and study of new molecules with dual, triple, or quadruple action targeting GLP-1, GIP, glucagon, and IGF-1 is important to cover as many pathogenic links of obesity with as few adverse effects as possible. Thus, currently, dual combinations of GLP-1 and GIP receptor agonists, such as Tirzepatid, or triple combinations of GLP-1, GIP, and glucagon receptor agonists, such as retatrutide, are under study or even available, as well as molecules with a dual mechanism of action, stimulating GLP-1 receptors and inhibiting GIP receptors, such as maridebart/cafraglutide, but additional studies are needed to evaluate their long-term effects and their effects in the pediatric population [93].

## 6. Future Directions

While GLP-1 agonists like liraglutide, semaglutide, and exenatide have demonstrated potential in treating childhood obesity, there remain a number of unanswered concerns regarding the optimal way to use them while understanding their wider effects. Targeted therapy, treatment combinations, impacts on growth and development, early intervention in children, and mental health outcomes are some of these concerns. Even if these treatments might be effective in preventing childhood obesity, there are still other unexplored pathways that could improve the understanding and use of these drugs. These options include innovative processes, combinatorial therapies, and long-term outcomes.

Current research predominantly focuses on adolescents aged 12 years and older; however, investigating the use of GLP-1 agonists in younger children (e.g., ages 6–11) with obesity might substantially expand the range of these therapies. Considering that childhood obesity can lead to early-onset insulin resistance and other metabolic disorders, early treatment may have long-term benefits. The effectiveness of GLP-1 agonists in treating pediatric obesity may vary based on individual genetic factors. Identifying genetic markers that predict which children will respond best to GLP-1 agonists could allow for personalized treatment plans. For instance, genetic variations in GLP-1 receptors or metabolic pathways could influence how well a child responds to treatment.

Additional research on metabolic phenotypes may elucidate distinct obesity subtypes (e.g., insulin resistance, dyslipidemia, or chronic inflammation) that may derive differential benefits from GLP-1 receptor agonist therapy. Metabolic syndrome is a domain that remains inadequately investigated in the pediatric population. Examining the use of GLP-1 agonists in pediatric patients with hypertension, dyslipidemia, hypertriglyceridemia, and abdominal obesity may provide insights into the potential of these drugs to mitigate cardiovascular illnesses in obese adolescents.

Further studies are needed to establish the optimal modulation of GIP receptors for the management of obesity and associated metabolic disorders, as well as to establish the indications and limitations of the GIP-targeting molecules for the pediatric population.

Improved long-term weight reduction and behavioral modifications may result from the combination of GLP-1 agonists with therapies that target mental health, behavior modification, and family support. Analyzing the combined impact of behavioral treatment and medication may improve adherence and provide longer-lasting results.

The long-term impact of GLP-1 receptor agonists on pediatric growth and development remains largely unknown. Considering that these medications may have effects on appetite regulation, dietary intake, and hormonal regulation, it is crucial to examine their effects on the pubertal development and bone health of children. For example, while GLP-1 agonists may lead to significant weight loss, it is essential to avoid nutritional deficiencies (e.g., calcium or vitamin D), particularly during critical developmental stages. Longitudinal studies are crucial to determine whether GLP-1 agonists affect pubertal velocity, bone density, and sexuality in teenagers, since these factors are significant at this developmental stage. The potential decrease in muscle mass associated with GLP-1 agonist medication and its consequences for pediatric populations requires additional research, especially considering the essential role of muscle development during growth.

The hormonal fluctuations typical of puberty may substantially influence enteroendocrine responses, particularly the release of the incretins GIP and GLP-1. The elevation of sex hormone levels (estrogens and testosterone), growth hormone, and insulin growth factor 1 (IGF-1) may affect the entero-insular axis [102]. These variables may alter the incretin response to meal consumption, influencing glucose homeostasis and the insulin response. Furthermore, insulin sensitivity fluctuates according to the stage of puberty, potentially affecting the efficacy of incretin-based therapy. To enhance obesity therapy in the pediatric population, more research is required to examine the dynamics of GIP and GLP-1 production relative to Tanner stages and the long-term impacts on muscle development, bone density, and sexual maturation.

The most current research recognizes the influence of microbiota in enteroendocrine cells’ gene expression, vesicle cycling, and hormone secretion (GLP, peptide YY), thereby directly impacting insulin regulation [103]. Animal studies show that obesity-related precocious puberty is accompanied by dysbiosis and lower short-chain fatty acid levels, indicating that these have a potential influence on the entero-insular and hypothalamic–pituitary–gonadal axes, linking microbiota, glucose homeostasis, and pubertal timing [104].

Several studies have indicated that genetic background could impact the entero-insular axis. Genetic determinants—including receptor polymorphisms (receptors of GLP1 and GIP), transcriptional regulators (*TCF7L2*), monogenic diabetes genes, and epigenetic modifications—profoundly influence the entero-insular axis in children [105]. These findings provide potential strategies for treating pediatric metabolic disturbances.

Future directions can refer to the identification of GLP1/GIP receptors and incretin pathway variants in pediatric populations to guide therapeutic strategies (e.g., predicting responses to GLP-1 receptor agonists), screening for genetic markers in at-risk children to facilitate early preventive measures, and targeting epigenetic changes (e.g., GLP1R methylation) to potentially modulate incretin axis responsiveness.

Lastly, future clinical trials should prioritize diverse pediatric populations, including various ethnic groups, socioeconomic backgrounds, and children with comorbidities, to improve the generalizability of their findings. Investigating these possibilities will advance the development of more effective, individualized, and sustainable treatments for childhood obesity, ultimately improving long-term health outcomes.

## 7. Conclusions

GLP-1RAs were initially developed for their antihyperglycemic characteristics, but they also have demonstrated benefits in weight control and cardioprotection. Although GLP-1 receptor agonists can result in decreases in body weight, BMI, and systolic blood pressure, the conducted studies highlight that these effects are typically moderate. Furthermore, the actual therapeutic potential of these drugs may be understated due to the small number of available trials and their limited periods. Heterogeneity in dosage schedules and inconsistent treatment protocol adherence are further concerns. All of these results indicate GLP-1 receptor agonists—in particular, semaglutide and liraglutide—as potentially effective pharmaceutical agents for treating childhood obesity, especially when combined with lifestyle modifications. However, the current data limitations and response variability, especially among children under 12, call for careful, evidence-based application and additional research in a variety of pediatric populations.

Although GLP-1 agonists have potential as a therapeutic alternative for obesity in children and adolescents, their use remains exploratory, necessitating more research to ascertain their long-term safety and effectiveness in pediatric cohorts. Liraglutide and semaglutide have shown efficacy in clinical studies, especially in teenagers aged 12 and above; nevertheless, healthcare practitioners must evaluate the possible benefits against the associated dangers, necessitating vigilant monitoring of adverse effects.

Treatment for childhood obesity, like any therapy, must be individualized and integrated into a holistic strategy that includes lifestyle changes, psychological support, and an emphasis on enhancing overall health and well-being.

## Figures and Tables

**Table 1:** Characteristics of the studies with Glucagon like peptide-1 agonists in pediatric populations.

Drugs	Study Author and Year	Study Design	Sample Size, (n)	Ages and BMI Eligible for Study	Study Duration	Target Dose	Study Primary Objective	Study Secondary Anthropometric Objectives
Liraglutide	Danne T. et al., 2017 [95] NCT01789086	Phase 1 randomized, placebo-controlled, double -blind, parallel-group	Total = 21 Liraglutide = 14 Placebo = 7	12–17 years BMI ≥ 30 kg/m^2^ and ≤45 kg/m^2^ BMI ≥ 95th percentile for age and sex	6 weeks and 5–14-day follow-up period	Week 1: 0.6 mg/day, Week 2: 1.2 mg/day, Week 3: 1.8 mg/day, Week 4: 2.4 mg/day, Weeks 5–6: 3.0 mg/day	Number of treatment emergent adverse events	- Change in BMI z-score - Change in body weight (kg)
	Fox C.K. et al., 2025 [96] NCT04775082 SCALE KIDS	Phase 3 randomized, placebo-controlled, double -blind, parallel-group	Total = 82 Liraglutide = 56 Placebo = 26	6–12 years BMI ≥ 95th percentile for age and sex	56 weeks and 26-week follow-up period	3.0 mg/day Week 1: 0.6 mg/day for children with weight ≥ 45 kg and 0.3 mg/day for those with weight < 45 kg, and increased the dose in 0.6 mg increments weekly	Relative change in BMI (%)	- Relative change in body weight (%) - % of subjects achieving ≥5%, 10% reduction in BMI - Change in BMI z-score - Change in BMI as percentage of the 95th percentile (%) - Change in waist circumference (cm)
	Kelly A.S. et al., 2020 [86] NCT02918279 SCALE TEENS	Phase 3 randomized, placebo-controlled, double-blind, parallel-group	Total = 251 Liraglutide = 125 Placebo = 126	12–17 years BMI ≥ 30 kg/m^2^ BMI ≥ 95th percentile for age and sex	56 weeks and 26-week follow-up period	Week 1: 0.6 mg/day, Week 2: 1.2 mg/day, Week 3: 1.8 mg/day, Week 4: 2.4 mg/day, Weeks 5–56: 3.0 mg/day	Change in BMI z-score	- % of subjects achieving ≥5%, 10% reduction in BMI - Change in BMI (Kg/m^2^, %) - Change in body weight (Kg, %) - Change in waist circumference (cm)
	Mastrandrea L.D. et al., 2019 [97] NCT02696148	Phase 1 randomized, placebo-controlled, double-blind, parallel-group	Total = 24 Liraglutide = 16 Placebo = 8	7–11 years BMI ≥ 30 kg/m^2^ and ≤45 kg/m^2^ BMI ≥ 95th percentile for age and sex	At least 7 weeks, and up to 6 optional treatment weeks, up to a maximum of 13 weeks.	Week 1: 0.3 mg/day Week 2: 0.6 mg/day, Week 3: 0.9 mg/day Week 4: 1.2 mg/day Week 5: 1.8 mg/day Week 6: 2.4 mg/day Weeks 7–13: 3.0 mg/day	Number of treatment-emergent adverse events	- Change in BMI z-score - Change in body weight (kg)
Exenatide	Weghuber D. et al., 2020 [98] NCT02794402	Phase 2 randomized placebo-controlled, double-blind, parallel-group	Total = 44 Exenatide = 22 Placebo = 22	10–18 years BMI z-score > 2.0 or age-adapted BMI > 30 kg/m^2^	24 weeks and 2-week follow-up period	2 mg/week	Change in BMI z-score	- Change in body weight (Kg, %) - Change in BMI (Kg/m^2^, %) - Change in BMI as percentage of the 95th percentile (%) - Change in waist circumference (cm)
	Kelly A.S. et al., 2012 [91] NCT00886626	Phase 2 randomized, crossover, controlled, open-label	Total = 12	8–19 years BMI ≥ 99th percentile for age and sex BMI ≥ 1.2 times the 95th percentile or BMI ≥ 35 kg/m^2^	6 months: 3-month control phase and 3-month drug phase	Month1: 5 mcg BID Months 2–3: 10 mcg BID	Change in BMI (Kg/m^2^)	- Relative change in BMI (%) - Change in body weight (kg, %)
	Kelly A.S. et al., 2013 [92] NCT01237197	Phase 2 randomized placebo-controlled, double-blind, parallel-group	Total = 26 Exenatide = 13 Placebo = 13	12–19 years BMI ≥ 1.2 times the 95th percentile or BMI ≥ 35 kg/m^2^	3-month randomization period and 3-month open-label extension period (all participants received exenatide)	**Randomization period:** Month 1: 5 mcg BID Months 2–3: 10 mcg BID **Open-label extension period** Month 1: 5 mcg BID Months 2–3: 10 mcg BID	Change in BMI (Kg/m^2^, %)	
Semaglutide	Weghuber D.et al., 2022 [89] NCT04102189 STEP TEENS	Phase 3 randomized, placebo-controlled, double-blind, parallel-group	Total = 201 Semaglutide = 134 Placebo = 67	12–17 years BMI ≥ 95th percentile for age and sex or BMI ≥ 85th percentile with ≥ 1 weight-related coexisting condition (hypertension, dyslipidemia, obstructive sleep apnea, or type 2 diabetes)	68 weeks and 7-week follow-up period	Week 1–4: 0.25 mg/week Week 5–8: 0.5 mg/week Week 9–12: 1.0 mg/week Week 13–16: 1.7 mg/week Week 17–68: 2.4 mg/week	Relative change in BMI (%)	- Change in BMI (Kg/m^2^) - Change in BMI z-score - Change in BMI as percentage of the 95th percentile (%) - % of subjects achieving ≥5% reduction in BMI - Change in body weight (Kg, %) - % of subjects achieving ≥5%, 10%, 15%, or 20% reduction in body weight - % of subjects achieving improvement in weight category - Change in waist circumference (cm)

BID, twice per day; BMI, body mass index.

**Table 2:** Efficacy anthropometric parameters in the studies with Glucagon like peptide-1 agonists in pediatric populations.

Drugs	Study Author and Year	Efficacy Parameters			
		Weight Loss Effect	BMI Outcome	BMI z-Score	Others
Liraglutide	Danne T. 2017 [95]	**Body weight change (kg):** Liraglutide: −2.55 Placebo: −1.85	NA	**BMI z-score change:** Liraglutide: −0.12 Placebo: −0.10	NA
	Fox C.K. 2025 [96]	**Body weight absolute change (kg):** Liraglutide:1.1 Placebo: 7.1 **Body weight relative change (%)** Liraglutide: 1.6 Placebo: 10.0	**BMI relative change (%):** Liraglutide: −5.8 Placebo: 1.6 **BMI as percentage of the 95th percentile (%)** Liraglutide: −14.0 Placebo: −0.4	**BMI z-score change:** Liraglutide: −0.7 Placebo: −0.3	**BMI reduction threshold (≥5%; ≥10%):** Liraglutide: 46.2%; 34.6% Placebo: 8.7%; 4.3% **Waist circumference (cm):** Liraglutide: −2.0 Placebo: 1.3
	Kelly A.S. 2020 [86]	**Body weight absolute change (kg):** Liraglutide: −2.26 ± 0.94 Placebo: 2.25 ± 0.98 **Body weight relative change (%)** Liraglutide: −2.65 ± 0.93 Placebo: 2.37 ± 0.95	**BMI absolute change:** Liraglutide: −1.39 ± 0.31 Placebo: 0.19 ± 0.33 **BMI relative change (%):** Liraglutide: −4.29 ± 0.88 Placebo: 0.35 ± 0.91 **BMI as percentage of the 95th percentile (%)** Liraglutide: −5.47 ± 1.2 Placebo: 0.77 ± 1.27	**BMI z-score absolute change:** Liraglutide: −0.23 ± 0.05 Placebo: −0.00 ± 0.05 **BMI z-score relative change (%):** Liraglutide: −8.32 ± 1.68 Placebo: −0.68 ± 1.74	**BMI reduction threshold (≥5%; ≥10%):** Liraglutide: 43.3%;26.1% Placebo: 18.7%;8.1% **Waist circumference (cm):** Liraglutide: −4.35 ± 0.85 Placebo: −1.42 ± 0.88
	Mastrandrea L.D. 2019 [97]	**Body weight absolute change (kg):** Liraglutide: −0.52 Placebo: 0.98	NA	**BMI z-score absolute change:** Liraglutide: −0.3 Placebo: −0.01	NA
Exenatide	Weghuber D. 2020 [98]	**Body weight absolute change (kg):** Exenatide: −0.5 Placebo: 2.5	**BMI absolute change (kg/m^2^):** Exenatide: −0.3 Placebo: 0.5 **BMI as percentage of the 95th percentile (%)** Exenatide: −0.2 Placebo: 0.0	**BMI z-score absolute change:** Exenatide: −0.1 Placebo: 0.0	**Waist circumference (cm):** Exenatide: −1.9 Placebo: 1.0
	Kelly A. S. 2012 [91]	**Body weight absolute change (kg):** Exenatide: −0.99 Control: 2.97 **Body weight relative change (%)** Exenatide: −1.2 Control: 2.68	**BMI absolute change (Kg/m^2^):** Exenatide: −0.9 Control: 0.84 **BMI relative change (%):** Exenatide: −2.57% Control: 1.72	NA	NA
	Kelly A. S. 2013 [92]	**Body weight absolute change (kg):** Exenatide: −2.9 Placebo: 0.32	**BMI absolute change (Kg/m^2^):** Exenatide: −1.18 Placebo: −0.04 **BMI relative change (%):** Exenatide: −2.9 Placebo: −0.15	NA	**Waist circumference (cm):** Exenatide: −2.04 Placebo: −1.01
Semaglutide	Weghuber D. 2022 [89]	**Body weight absolute change (kg):** Semaglutide: −17.0 Placebo: 2.3 **Body weight relative change (%)** Semaglutide: −16.3 Placebo: 2.6	**BMI absolute change (Kg/m^2^):** Semaglutide: −6.5 Placebo: 0.1 **BMI relative change (%):** Semaglutide: −17.9% Placebo: 0.6% **BMI as percentage of the 95th percentile (%)** Semaglutide: −26.7% Placebo: −4.4%	**BMI z-score absolute change:** Semaglutide: −1.2 Placebo: −0.1	**-** **% of subjects achieving ≥5% reduction in BMI** Semaglutide: 78.2% Placebo: 20.7% **-** **% of subjects achieving ≥5%, 10%, 15%, or 20% reduction in body weight** Semaglutide: 75.6%; 64.7%; 56.3%; 39.5% Placebo: 15.5%; 8.6%; 5.2%; 3.4% **-** **% subjects achieving improvement in weight category** Semaglutide: 71.8% Placebo: 21.0% **-** **Waist circumference (cm):** Semaglutide: −13.9 Placebo: −0.1

BMI, body mass index; NA, not available.

**Table 3:** Safety parameters in the studies with Glucagon like peptide-1 agonists in pediatric populations.

Drugs	Study Author and Year	Safety Parameters: GLP-1 Agonist vs. Control (Percent of Participants Experiencing at Least One Adverse Event Episode)				
		Any Treatment-Emergent Adverse Events (TEAEs)	Gastrointestinal Adverse Events	Adverse Events that Led to Treatment Discontinuation	Treatment-Emergent Serious Adverse Events	Others Adverse Events
Liraglutide	Danne T., 2017 [95]	Total: 100% vs. 57.1% Total TEAEs possibly/probably related to investigational product: 14.3/92.9% vs. 0.0%	Total 85.7% vs. 28.6% Total GI AEs possibly or probably related to investigational product: 78.6% vs. 0.0% of which: Nausea 50.0% vs. 0.0% Vomiting 34.4% vs. 0.0% Diarrhea 22.4% vs. 0.0% Abdominal pain 8.0% vs. 0.0%	0.0% vs. 0.0%	0.0% vs. 0.0%	**Hypoglycemic episodes:** Confirmed: 14.3% vs. 0.0% ADA: 57.1% vs. 14.3% **Nervous system disorders**: 50.0% vs. 14.3%
	Fox C. K., 2025 [96]	89.3% vs. 88.5%	Total 80.4% vs. 53.8%	10.7% vs. 0.0%	12.5% vs. 7.7%	**Psychiatric disorders**: 10.7% vs. 11.5%
	Kelly A.S., 2020 [86]	88.8% vs. 84.9%	Total 64.8% vs. 36.5% Nausea 42.4% vs. 14.3% Vomiting 34.4% vs. 4.0% Diarrhea 22.4% vs. 14.3% Abdominal pain 8.0% vs. 8.7%	10.4% vs. 0.0%	2.4% vs. 4.0%	**Pancreatitis**: 0.8% vs. 0.0% **Hypoglycemia**: 20.8% vs. 14.3% **Psychiatric disorders**/depression/suicidal ideation/completed suicide: 10.4/4.0/0.8/0.8% vs. 14.3/2.4/0.8/0.0%
	[97]	Total: 56.3% vs. 62.5% Total TEAEs possibly/probably related to investigational product: 31.3/18.8% vs. 12.5/12.5%	Total 37.5% vs. 12.5% Nausea 12.5% vs. 0.0% Vomiting 25.0% vs. 0.0% Diarrhea 6.3% vs. 0.0% Abdominal pain 6.3% vs. 0.0%	0.0% vs. 0.0%	0.0% vs. 0.0%	**Hypoglycemic episodes:** ADA: 25.0% vs. 12.5% **Nervous system disorders**: 18.8% vs. 50.0%
Exenatide	Weghuber D. 2020 [98]	Total TEAEs possibly/probably related to investigational product: 81.8/68.2% vs. 13.6/13.6%	Total 81.8% vs. 45.5%	4.5% vs. 0.0%	0.0% vs. 0.0%	**Hypoglycemic episodes:** ADA: 0.0% vs. 0.0% **Nervous system disorders**: 72.7% vs. 59.1% **Psychiatric disorders**: 4.6% vs. 9.1%
	Kelly A. S., 2012 [91]	Total: 36.36% vs. 0.0%	Nausea 36.36% vs. 0.0% Vomiting 27.27% vs. 0.0% Abdominal pain 27.27% vs. 0.0%		0.0% vs. 0.0%	**Pancreatitis**: 0.0% vs. 0.0% **Hypoglycemia**: 0.0% vs. 0.0%
	[92]	NA	Nausea 62% vs. 31% Vomiting 31%vs 8% Diarrhea 8% vs. 31% Abdominal pain 15.0% vs. 23.0%	NA	0.0%vs 0.0%	**Pancreatitis**: 0.0% vs. 0.0% **Hypoglycemia**: 0.0% vs. 0.0% **Nervous system disorders**: 23.08% vs. 46.15%
Semaglutide	[89]	79% vs. 82%	Total 61.7% vs. 41.8% Nausea 42.1% vs. 17.9% Vomiting 36.0% vs. 10.4% Diarrhea 21.8% vs. 19.4% Abdominal pain 15.04% vs. 5.97%	5% vs. 4%	11.28% vs. 8.96%	**Pancreatitis**: 0.0% vs. 0.0% **Hypoglycemia**: 0.0% vs. 0.0% **Psychiatric disorders**: 0.75% vs. 0.0%

TEAEs, Treatment-emergent adverse events; GI, gastrointestinal; AEs, adverse events; ADA, American Diabetes Association; GLP-1; NA, not available.

## Abbreviations

The following abbreviations are used in this manuscript:

BMI	Body mass index
GLP-1	Glucagon-like peptide-1
GIP	Gastric inhibitory polypeptide
DPP-4	Dipeptidyl peptidase 4
cAMP	Cyclic adenosine monophosphate
HOMA-IR	Homeostasis Model Assessment of Insulin Resistance
OGTT	Oral glucose tolerance test
TM	Transmembrane
BID	Twice per day
NA	Not available
GI	Gastrointestinal
AE	Adverse event
ADA	American Diabetes Association
TEAE	Treatment-emergent adverse event
